# Stable isotope analyses (*δ*^15^N, *δ*^34^S, *δ*^13^C) locate early rye cultivation in northern Europe within diverse manuring practices

**DOI:** 10.1098/rstb.2024.0195

**Published:** 2025-05-15

**Authors:** Frank Schlütz, Felix Bittmann, Susanne Jahns, Sonja König, Lyudmila Shumilovskikh, Michael Baumecker, Wiebke Kirleis

**Affiliations:** ^1^ Institute of Prehistoric and Protohistoric Archaeology, Kiel University, Kiel, Schleswig-Holstein, Germany; ^2^ Lower Saxony Institute for Historical Coastal Research, Wilhelmshaven, Niedersachsen, Germany; ^3^ Institute of Geography, GEOPOLAR, University of Bremen, Bremen, Germany; ^4^ Brandenburgisches Landesamt für Denkmalpflege und Archäologisches Landesmuseum, Zossen, Brandenburg, Germany; ^5^ Ostfriesische Landschaft, Aurich, Niedersachsen, Germany; ^6^ Department of Palynology and Climate Dynamics, Georg-August-Universität Göttingen, Göttingen, Niedersachsen, Germany; ^7^ Albrecht Daniel Thaer-Institut für Agrar- und Gartenbauwissenschaften, Humboldt-Universität zu Berlin, Berlin, Germany

**Keywords:** *Secale cereale*, fertilization, archaeobotany, Middle Ages, terps, triple stable isotopes

## Abstract

Stable isotopes provide insights into the early history of rye cultivation from the Migration period to the late Medieval period (fourth to fifteenth centuries CE). Manuring shows high intensity and diversity throughout. Rye as an undemanding crop resistant to drought was cultivated on nutrient-poor sandy soils to a limited extent only. It became a dominant crop owing to its integration into an existing labour-intensive manuring system mainly based on stable dung. Modern experiments demonstrate that the effect of manuring on cereal *δ*^15^N is strongly mediated by the soil substrate. Conspicuously low *δ*^34^S values can indicate additional manuring with peat. The Δ^13^C values suggest that the best harvests were achieved on dwelling mounds close to the sea and that relatively poor harvests resulted on fields on dry, sandy soils. Because the mounds were flooded with salt water during winter storm surges, the crop cultivated there might have been summer rye. Winter rye became the dominant crop in Germany around 1000 CE and continued to be until the mid-twentieth century. Intensive manuring allowed for high yields, which facilitated the emergence of village communities and towns and stable political and religious power systems.

This article is part of the theme issue ‘Unravelling domestication: multi-disciplinary perspectives on human and non-human relationships in the past, present and future’.

## 1. Introduction

Rye is a latecomer as a staple crop in European prehistory. Its domestication to date has been a two-way process of interaction between humans and the environment, including between species emerging as strong competitors and technological innovations in agriculture, such as the invention of the mouldboard plough in the last centuries BCE. Using stable isotope analysis, we examine another aspect of arable farming practice, namely manuring, and discuss the extent to which fertilization influenced the adoption of rye as a major crop from medieval times onwards. This provides a much more holistic understanding of early agriculture and domestication, as the link between plant and animal husbandry can be crucial [1].

### (a) Prehistory of rye domestication and its relevance within agro-ecosystems

Rye was only occasionally cultivated in the Levant during the Aceramic Neolithic or even earlier [2,3]. It was introduced to Europe as an arable weed infesting the fields of barley and wheat in the Early Neolithic [4,5]. The number of archaeobotanical finds in Europe relating to the Bronze Age is higher, but it was not until the Iron Age that there was intentional cultivation as a staple food, as indicated by the first rye-dominated assemblages [4,6,7].

This post-Neolithic domestication took place independently at various locations in Europe and at the northernmost edge of southwestern Asia during the centuries around the turn to the Common Era [4,6,8,9]. The transition from rye being a weed admixture to it being a cereal in its own right was discussed by Behre [4] against the background of the low soil and climatic requirements of rye, in conjunction with climate deterioration and harvesting close to the ground, which had become possible following the adoption of iron sickles.

In the sandy areas of northern Germany, a labour-intensive, permanent system of rye cultivation called *Ewiger Roggenbau* (eternal rye cultivation) developed at the start of the second millennium CE. This included *Plaggenwirtschaft* (sod agriculture)—cutting heath sods, using these for stable bedding, and later depositing the resulting soil-and-dung mixture on certain plots to allow permanent crop cultivation. By this process, the ground surface, enriched by plaggen manure, rose above that of the surrounding landscape and the steady influx of nutrients allowed permanent cultivation of rye and harvesting of the grains and the straw. A one year fallow phase was necessary approximately every 10 years or sometimes even longer [10–12].

### (b) Manuring and stable isotope analyses

Compared with the natural baseline, the concentrations of nitrogen and of the heavy nitrogen isotope ^15^N are elevated in dung. Consequently, cereals cultivated on manured soils can exhibit remarkably high *δ*^15^N values [13,14]. Research on isotopes of archaeological rye is sparse, but ancient rye grains with elevated ^15^N content are known from southern Scandinavia as early as the fifth to sixth centuries CE [15], predating the plaggen-based medieval system of rye cultivation by half a millennium [12]. This raises the question: to what extent was early European rye cultivation integrated into labour-intensive cultivation systems [16] and to what extent did it involve targeted manuring? Here, we rethink the early northern European cultivation of rye based on new isotope measurements of *δ*^15^N, *δ*^34^S and *δ*^13^C on rye grains from settlements in eastern Germany and on the German North Sea coast, dating to the Migration, Slavic and Medieval periods (table 1). While comparing isotope data on rye from manured and unmanured cultivation experiments, we also discuss the variable influence of manuring intensity and soil type on the *δ*^15^N values of cereal grains. Together with isotope data from the literature [15,17–19], this gives a fairly clear idea of how diverse manuring practices led to domesticated rye becoming one of the dominant crops in great parts of northern Europe for more than 1500 years, until the mid-twentieth century [20].

**Table 1.** Archaeological periods of northwestern and ^a^eastern Germany with date range in years CE.

period	years CE
Roman period	1−350
Migration period	350−500
early Medieval period	500−1000
Slavic period^a^	600−1200
high Medieval period	1000−1300
late Medieval period	1300−1500

## 2. Material

### (a) Rye from modern cultivation experiments

**Figure 1.**
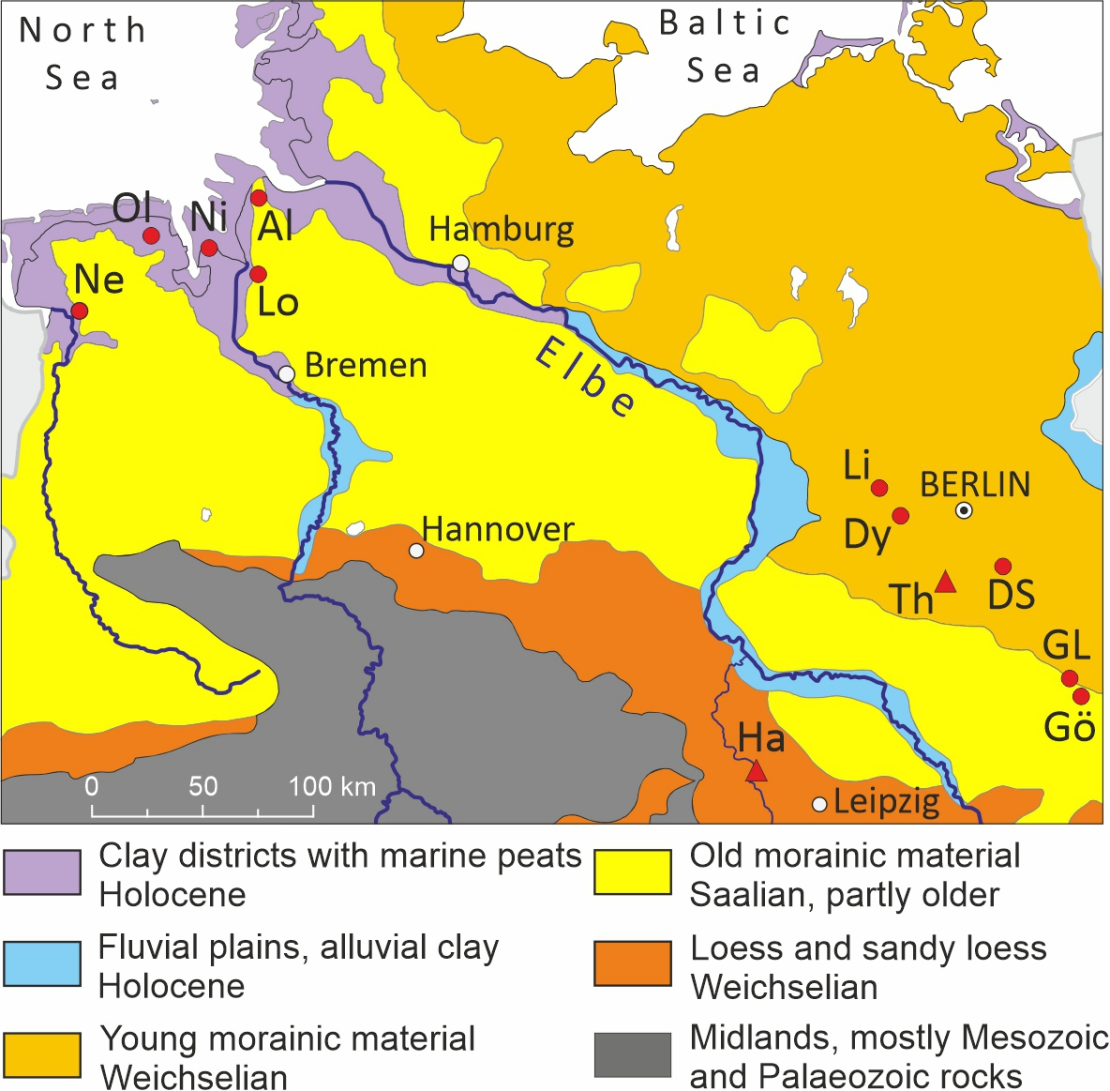
Soil regions of northern Germany with the sites Neermoor (Ne), Oldorf (Ol), Niens (Ni), Loxstedt (Lo), Altenwalde (Al), Lietzow (Li), Dyrotz (Dy), Diepensee (DS), Groß Lübbenau (GL), Göritz (Gö), Thyrow (Th), Halle (Ha) (map source [21]).

To establish the impact of manuring on *δ*^15^N values in rye grains, we studied grains from two experimental agricultural sites in eastern Germany, at Thyrow and Halle/Saale (figure 1). Harvesting included the straw, resulting in a high nutrient extraction from the soil. At Thyrow, 30 km southwest of Berlin, the Static Nutrient Deficiency Experiment, on slightly silty sands with low organic content, started in 1937. The soil, an Albic Luvisol, which developed from rust-coloured forest soils, has low water-holding capacity and is typical of the moraine landscape of eastern Germany. The mean annual temperature and precipitation are approximately 9.7°C and 533 mm (1991−2020), respectively, and there was a water deficit in summer. The cultivation of winter rye as monoculture without rotation started in 1998, including on an unmanured plot and on a plot manured with an average 15 t ha^−1^ yr^−1^ of dung [22]. At Halle/Saale, the Eternal Rye Experiment started in 1878. Winter rye has been cultivated since then in monoculture without crop rotation. The base of morainic material is here covered by sandy loess, from which a Luvic Chernosem developed [23]. Mean annual temperature and precipitation are 9.6°C and 516 mm (1981−2010), respectively. The fields have been manured with 12 t ha^−1^ yr^−1^ of stable dung from 1878 to the present day and from 1893 to 1952, respectively [23,24]. *δ*^15^N values have been measured from a total of 80 grains (20 from each level of manuring) harvested in 2017 and artificially charred.

### (b) Rye grains from archaeological contexts

The analysed ancient cereal grains derive from archaeological excavations conducted during the past decades in the Lower Saxony part of the North Sea coast, in northern Germany, and in the state of Brandenburg, in eastern Germany, and—as a comparator—from excavations in the Caucasus Mountains in Georgia. The rye from the German coastal area originates from two geologically and hydrologically different landscapes—the wet marshlands of the German clay district and the dry Geest (upland), respectively. While the subsoil of the flat clay district consists mostly of peats and marine clays formed during the Holocene, that of the hilly Geest is characterized by sands and gravels from the Saalian (or penultimate) glaciation.

In northern Germany, the German clay district (*Marsch*, or marshland) has been inhabited from the Late Bronze Age (*ca* 800 BCE) onwards. During phases of low marine influence, habitation took place on flat, since dried-up land; during phases of increased marine influence, it took place on artificial dwelling mounds (or terps; German: *Wurten* or *Warften*) erected on the flat ground [25,26]. In both settings, the houses were divided into a living space and a stable part [27]. Following a rise in sea level, manure, drifted organic material and marine clay were stacked in alternating layers to heighten the dwelling mounds, and over time, small mounds with single farmsteads or few houses often fused to larger village mounds [28]. The *Wurt* **Niens** (3–4 m a.s.l.) is occupied today by a farm, that of **Oldorf** (up to 5 m a.s.l.), in Frisia, by a village (electronic supplementary material, table s1). Niens was founded on the elevated embankment of a former tideway surrounded by since dried-up marine sediments in the north of what is today the Butjadingen peninsula [27].

The early Medieval colonization of Niens started on level ground at the end of the seventh century CE and ended in the first half of the ninth century (table 2). At the start, freshwater conditions prevailed, but with the rising sea level, these turned into marine conditions, and a dwelling mound was erected as protection against winter storm tides [27,30]. Plough marks and hoof prints of cattle from the initial colonization are preserved below parts of the mound, testifying to the economic activities. The remains of rye analysed have been ^14^C dated to the seventh to eighth centuries (electronic supplementary material, table s2). As one of the oldest Frisian settlements, Oldorf was founded in the first half of the seventh century. Like at Niens, the economy was mainly based on livestock breeding, and the mound partially covers former arable land. The medieval settlement ended in the eleventh century [27]. Rye found here has been ^14^C dated to the ninth century. **Loxstedt** and **Altenwalde** are settlements situated on the Geest ridge Hohe Lieth (approximately 25 m a.s.l. at Altenwalde), surrounded by the clay district to the north, west and east. Loxstedt, south of Bremerhaven, was an agricultural village of more than a dozen long houses with incorporated stables and approximately 100 pit houses of the fourth to sixth centuries [31]. The archaeobotanical material from Altenwalde, including rye, was found in several pits during an emergency excavation, some also containing pottery of the fourteenth century [32]. Barley from the same context was dated to the thirteenth to fourteenth centuries. The remains of rye from **Neermoor** are from a late Medieval castle built on the lower part of a flat Geest slope in the thirteenth century and destroyed no later than 1409 CE. The excavated castle measured 70 × 70 m and was surrounded by a 6−8 m wide water-filled moat [33,34]. Adjoining it to the west are large areas of raised bog with *Sphagnum* peats covered by brackish clays [35].

**Table 2.** Overview of the investigated archaeological sites. (Ages are given for the archaeological site or the ^a^calibrated ^14^C dates of cereals (electronic supplementary material, table s2). LBK, Linearbandkeramik (*Triticum dicoccum*) here 5200−5000 cal. BCE [29].)

site name	abbreviation	region	site type	age (centuries CE)
Niens	Ni	marshland, clay district	terp, farm	7−8^a^
Oldorf	Ol	marshland, clay district	terp, settlement	7−9^a^
Loxstedt	Lo	Geest, old moraine	village	4−6
Altenwalde	Al	Geest, old moraine	village	13−14^a^
Neermoor	Ne	Geest, old moraine	fortification	13−15
Lietzow	Li	eastern Germany, young moraine	village	LBK
Göritz	Gö	eastern Germany, old moraine	village	3−5
Groß Lübbenau	GL	eastern Germany, old moraine	fortification	9−10
Dyrotz	Dy	eastern Germany, young moraine	village	10−13
Diepensee	DS	eastern Germany, young moraine	village	13−14
Dariali Fort	DF	mesozoic rocks, Georgia	fortification	4−5

The material from eastern Germany derives from five excavations in different parts of the county of Brandenburg, including around Berlin [36]. The geological subsoil is dominated by morainic sediments of the last and the second-to-last glaciation. While the older material, from the Saalian glaciation, has become leached over time, the younger material, from the Weichselian glaciation, is richer in nutrients. Some of the field investigations were carried out ahead of site destruction by opencast lignite mining [37,38] and expansion of the Berlin-Brandenburg airport [39].

**Göritz** is situated on substrates of the Saalian glaciation [40]. It represents a late Roman period settlement continuously inhabited for more than 200 years, starting in the mid-third century CE. It yielded remains of 150 houses and rich finds of rye grains and cattle bones [37]. The weed assemblage suggests regular and intensive cultivation that probably included crop rotation and fallowing and thus some kind of manuring by grazing animals [36]. The rye from **Groß Lübbenau** is part of a rye-dominated mass find of cereal from a Slavic castle dated to the ninth to tenth centuries that became charred during a major fire that destroyed the castle [38]. In the high Medieval Anger village of **Diepensee** (thirteenth century to 1375 CE at the latest), rye dominates as well. The occurrence of bread wheat (*Triticum aestivum*) and its associated weeds and the low proportion of millet (*Panicum miliaceum*) point to good soil conditions [36,39,41]. The soil substrate around Diepensee is built of glacial sediments of the Weichselian (LBGR) [40]. Archaeozoological and genetic studies have demonstrated the great importance of horse husbandry [42]. At the Slavic site of **Dyrotz** (late tenth or early eleventh to early thirteenth century), also located in an area with Weichselian deposits, finds of rye were rare, while finds of wheat were quite common [36,43,44].

To better understand the rye isotopic values, we compare them with values of emmer (*Triticum dicoccum*) of the Linearbandkeramik (LBK) period from a pit at **Lietzow** [29,45] and dated to the late sixth millennium BCE. The geological substrate consists of Weichselian deposits, and there are peats in a moist depression nearby [40]. We also compare them with rye co-dominated finds from the earliest occupation phase at Dariali Fort, in the Caucasus, radiocarbon-dated to the mid-fourth to early fifth century. Situated at 1350 m a.s.l. on a steep valley slope, the fort controls a pass from southwestern Asia, in the south, to eastern Europe, in the north. Late Medieval terraces up to 1950 m a.s.l. point to local cereal cultivation, and this is evidenced by archaeobotanical findings. An abundance of animal bones in the Dariali Fort indicates animal husbandry and the availability of dung for agricultural practices [46].

## 3. Methods

### (a) Pre-treatment

The charred archaeobotanical grains were cleaned with 6 M HCl overnight to remove mineral adherents and then washed and stored overnight in demineralized water [47–49]. For Oldorf, where calcareous layers of marine clay prevailed, an additional subsample was treated with demineralized water only and measured for *δ*^13^C and *δ*^15^N. For the *δ*^13^C values, an ANOVA test for equal means performed with the software Past v. 4.11 [50] indicates statistically significant differences (*p* < 0.01), and therefore only the 6 M HCl data are considered further. For *δ*^15^N (*p* = 0.63), values of both treatments are combined. For Neermoor, an additional three subsamples were cleaned for approximately 1 min with 6 M HCl, 10% HCl or overnight with demineralized water. The ANOVA test including the 6 M HCl overnight samples showed insignificant statistical differences (*δ*^13^C, *p* = 0.33; *δ*^15^N, *p* = 0.40; *δ*^34^S, *p* = 0.26), and the results of all subsamples are therefore combined. The modern grains (Halle, Thyrow) were charred in small, open porcelain crucibles at 250°C for 5 h. All values in the text, figures and tables refer to charred grains and are uncorrected for charring [51]. All grains were individually weighed and then mortared, and the powder was weighed into tin capsules. For some measurements, 2–10 grains were necessary to reach the needed sample weight (electronic supplementary material, table s3). As temperature, duration of firing (possible) contact with atmospheric oxygen and the moisture content of the grains all affect the charring process and thus preservation, the best preserved grains were selected for isotopic measurements to reduce the influence of these factors on the results.

### (b) Stable isotope measurements

For the measurement of the *δ*^13^C and *δ*^15^N at the isotope laboratory of the GeoZentrum, Erlangen, Germany, a Flash EA 2000 elemental analyser connected online to a Thermo Finnigan Delta V Plus mass spectrometer was applied. Additional samples were submitted to the Centre for Isotope Sciences, Glasgow, UK, for analysis of *δ*^34^S, *δ*^13^C and *δ*^15^N using a Thermo Fisher Scientific Delta V Plus Isotope-ratio mass spectrometry with an IsoLink Flash HT element analyser. Results are calibrated against the standards USGS 40, 41, MSAG2, M2, SAAG2 and reported as ‰ relative to VPDB for carbon, atmospheric air for nitrogen and VCDT for sulfur. The standard deviation was less than ±0.2‰ for *δ*^13^C, ±0.2‰ for *δ*^15^N and ±0.7‰ for *δ*^34^S.

### (c) Radiocarbon dating

Three grains were dated by the AMS Radiocarbon Laboratory at Adam Mickiewicz University, Poznań, Poland, and the resulting age data were calibrated using OxCal v. 4.4 with the IntCal20 calibration curve [52,53] (electronic supplementary material, table s2).

### (d) Calculation of relative grain yield from ^13^C content

**Figure 2.**
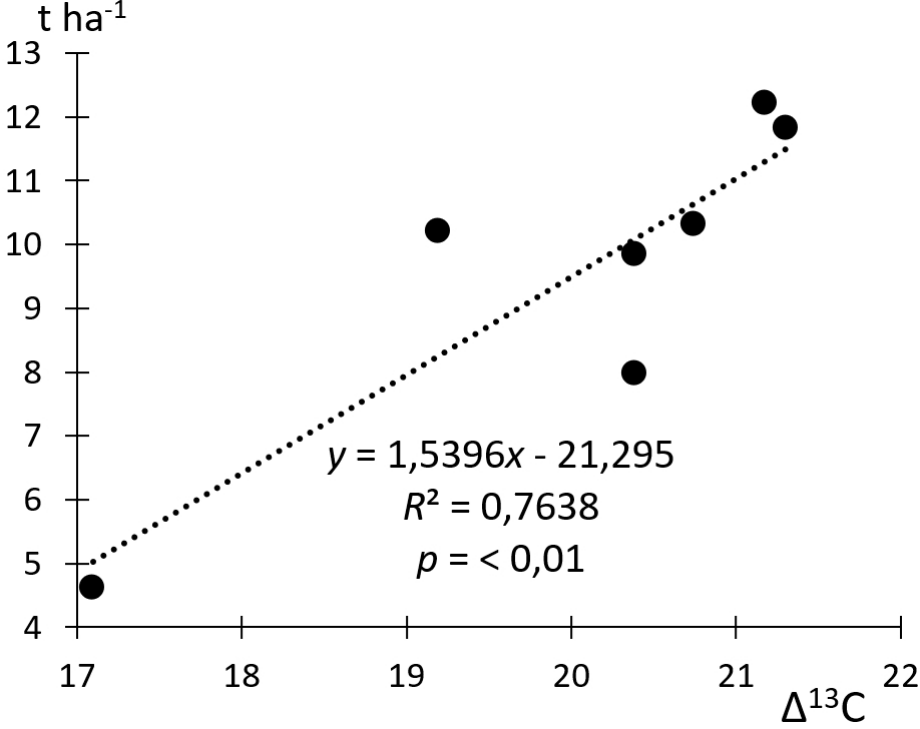
Relationship of rye yield to grain Δ^13^C in modern experiments (compare [58–60]).

The grain yield and ^13^C content are directly controlled by water availability. Under humid conditions with high yields, wide-open stomata increase the influx of CO_2_ into the plants, leading to a higher rate of carbon uptake and starch synthesis and consequently to a lower ^13^C content and a higher Δ^13^C in the grains. During water deficit, a reduced fractionation effect leads to a lower Δ^13^C value [54] . In this way, the ^13^C content of cereal grains is a proxy for the hydrological conditions during the summer months, when the grains develop, and is closely related to yield [51,55]. The conversion to Δ^13^C values calculates the changing atmospheric ^13^C content over time [56,57]. Araus *et al*. [56] developed a ^13^C-based calculation of yields of archaeological wheat and barley grains for the Mediterranean region. Because formulas for absolute yield amounts [56] rest on several arguable assumptions, we present here a formula for relative yields (ry) based on water deficit experiments with rye on loamy sands in northeastern Germany and Poland [58–60]. The results imply that a water shortage accounting for a Δ^13^C decrease of 1‰ results in a yield reduction of approximately 12.2% (figure 2).

## 4. Results

### (a) *δ*^15^N of modern rye

**Figure 3.**
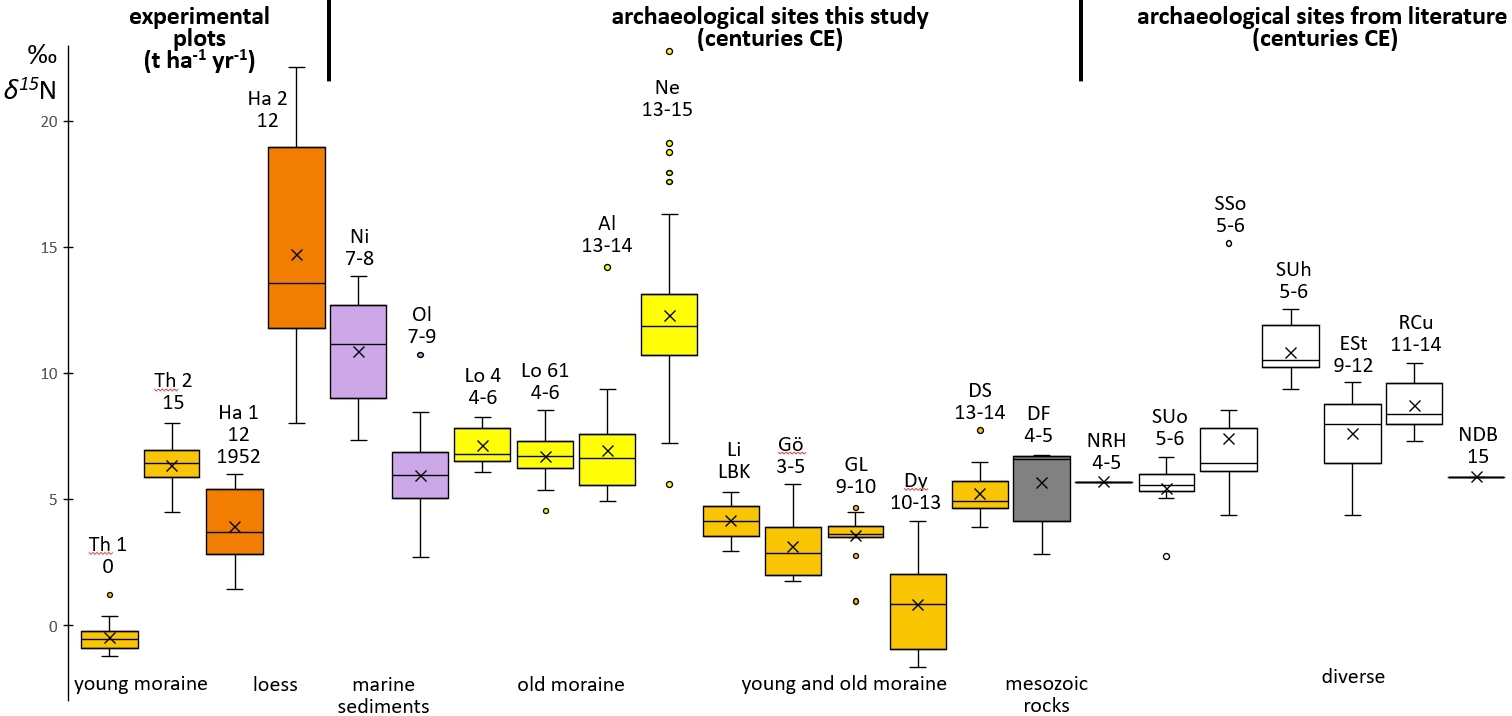
*δ*^15^N box plots of experimental sites (Th, Ha), *Wurten* (Ni, Ol), the Geest (Lo, Al), eastern Germany (Li to Dy), Dariali Fort (DF) in Georgia, and from literature from The Netherlands (Raalte‐Heeten (NRH); Deventer Burseplein (NDB)), Sweden (Uppåkra oven, Suo; Stanstorp oven, SSo; Uppåkra house 11, SUh), England (Stafford, ESt), Romania (Cunești, RCu) [15,17–19]. For Th and Ha, the numbers refer to the amount (t ha^−1 ^ yr^−1^) and last year (1952) of manuring. For the archaeological sites, the numbers represent the age of the archaeological material in centuries CE; LBK, Linearbandkeramik.

The unmanured rye from the sandy soils of Thyrow exhibits *δ*^15^N values around −0.5‰ (figure 3; table 3), which is close to the 0.0‰ *δ*^15^N of the atmosphere. Therefore, the N of this rye may originate initially from, among other things, nitrogen fixation by cyanobacteria or symbiotic nodule bacteria of legumes [61,62]. The manured rye shows much higher *δ*^15^N values, of around 6.3‰. Thus, in rye on sandy soils, a common pre-industrial level of manuring of 15 t ha^−1 ^yr^−1^ dung causes a clear increase in *δ*^15^N by some 7‰.

**Table 3.** Statistical characteristics of the *δ*^15^N for the 2017 rye harvest of recent experiments (Thyrow, Halle) and the cereals from the archaeological sites included in this study, including the minimum (min.), maximum (max.), mean, and standard deviation (s.d.) of *δ*^15^N, as well as the number (*n*) of measured grains.

site	abbreviation	sample origin	taxon	min.	max.	mean	s.d.	*n*
Thyrow	Th 1	plot 0 t ha^−1 ^ yr^−1^	rye	−1.2	1.2	−0.5	0.58	20
Thyrow	Th 2	plot 15 t ha^−1 ^ yr^−1^	rye	4.5	8.0	6.3	0.90	20
Halle	Ha 1	plot 12 t ha^−1 ^ yr^−1^ (1952)	rye	1.5	6.0	3.9	1.39	20
Halle	Ha 2	plot 12 t ha^−1 ^ yr^−1^	rye	8.0	22.1	14.7	4.16	20
Niens	Ni	*Wurt*	rye	7.4	13.9	10.8	2.03	14
Oldorf	Ol	*Wurt*	rye	2.7	10.7	5.9	1.40	47
Loxstedt 4	Lo 4	Geest	rye	6.1	8.3	7.1	0.70	21
Loxstedt 61	Lo 61	Geest	rye	4.6	8.5	6.7	0.81	24
Altenwalde	Al	Geest	rye	4.9	14.2	6.9	2.08	20
Neermoor	Ne	Geest	rye	5.6	22.8	12.3	2.62	90
Lietzow	Li	eastern Germany	emmer	2.9	5.3	4.2	0.71	18
Göritz	Gö	eastern Germany	rye	1.7	5.6	3.1	1.21	18
Groß Lübbenau	GL	eastern Germany	rye	1.0	4.7	3.6	0.88	14
Dyrotz	Dy	eastern Germany	rye	−1.6	4.1	0.8	2.00	6
Diepensee	DS	eastern Germany	rye	3.9	7.7	5.2	0.91	20
Dariali Fort	DF	Caucasus, Georgia	rye	2.8	6.8	5.7	1.67	5

On the loess soils of Halle, the rye manured from 1878 until 1952 (12 t ha^−1 ^ yr^−1^ dung) shows a mean value of 3.9‰, which is below the *δ*^15^N level of the manured rye from the Thyrow experiment. The highest mean, of 14.7‰, as well as the most variable *δ*^15^N, with values from 8.0 to 22.1‰, is exhibited by the rye manured continuously since 1878, pointing to the great influence of an additional 65 years of manuring. The more intensively manured plot of Thyrow has lower δ15N values. This seems to indicate that having loess as the initial soil substrate has a large positive influence on the *δ*^15^N.

### (b) Influence of manuring and soil quality on the *δ*^15^N

Rye from the loess soils of Halle manured by 12 t ha^−1 ^ yr^−1^ has a much higher *δ*^15^N than the more intensively manured rye from the sandy soils of Thyrow. This result is unexpected and not in line with a scale for manure intensity established by Bogaard *et al*. [14], widely accepted in archaeology and combined *δ*^15^N results from agricultural experiments in northern Europe. To summarize, in this standard, *δ*^15^N values below 3‰ stand for no, low or former manuring; values of up to approximately 6‰ stand for medium (10–15 t ha^−1 ^ yr^−1^) manuring; and values above 6‰ stand for high (35 t ha^−1 ^ yr^−1^) manuring. Our results from the modern cultivation plots bring these cut-off values from the literature into question. The standard seems applicable for the manured and the unmanured rye from the sandy soils of the Thyrow experimental plots but not for the rye from the loess soils of the Halle experimental plots, as the medium-manured rye from Halle exhibits a *δ*^15^N that corresponds to high manure application following the literature. In addition, the *δ*^15^N values of rye grown on plots last manured decades ago, in 1952, correspond not to low but to medium manure application following the literature. Obviously, besides manuring practices, soil quality also has a strong effect on the isotopic composition. As shown by Schlütz *et al*. [1], the natural baseline for *δ*^15^N of cereals from loess can in some landscapes be around 6‰.

### (c) *δ*^15^N of ancient rye

The archaeological isotopic data for northern Germany with the earliest dates are from the sandy Geest site Loxstedt (fourth to sixth centuries CE). The mean *δ*^15^N of the two samples are 6.7 and 7.1‰ (figure 3). The *δ*^15^N of the much younger rye from Altenwalde (thirteenth to fourteenth centuries), also from the sandy Geest, at 6.9‰, is close to the rye of Loxstedt. Both sites from the sandy Geest exhibit values that are much higher than those of the unmanured rye from the Thyrow experiment but close to those of the rye from the loess soils of Halle manured with 15 t ha^−1 ^ yr^−1^ dung . Therefore, it seems that rye has been cultivated on intensively manured fields since the Migration period or shortly thereafter, well before the *plaggen* soil system started in high Medieval times. The slightly wider *δ*^15^N range from Altenwalde may illustrate that the archaeological grains originated from more than one yield, thus from different fields or years [13] or from fields with a long history of manuring, like in the Halle experiment.

The *δ*^15^N range of the rye from the *Wurt* Oldorf (ninth century) falls within that of Loxstedt and Altenwalde, whereas the values from the *Wurt* Niens (seventh to eighth centuries) exhibit a much higher mean. As dwelling mounds were partly built with stable dung, the high *δ*^15^N values of Niens may point to the cultivation of rye, probably in small, garden-like plots, directly on the mounds. In addition to dung, the high *δ*^15^N could partly originate from material collected from drift lines at the seashore [63]. Flooding by storm surges in winter makes it likely that rye was cultivated in summer, after rain had diluted any salt residues of the winter storm surges. The rye *δ*^15^N of Neermoor, situated on the Geest close to the transition to the marshland, displays the highest and most diverse ^15^N enrichment of all archaeological sites. The Neermoor values evidence soils rich in ^15^N, such as those at Niens and even higher. Stable dung was probably a prominent source of ^15^N; however, as discussed below, the *δ*^34^S points to an additional source.

The archaeobotanical rye from the sandy area east of the river Elbe exhibits *δ*^15^N values clearly higher than those of the unmanured modern rye from Thyrow. The ^15^N enrichment of the medieval manuring at Diepensee was already equivalent to approximately 15 t ha^−1 ^ yr^−1^ of stable dung manuring. Some of the ancient rye from Göritz points to quite intensive manuring 1500 years ago, and the rye from Groß Lübbenau points to the same for the following Slavic period. However, this high *δ*^15^N level is not new, as it is already seen in the Neolithic emmer from Lietzow. When we compare the values with the rye from the Thyrow experiments, we see that some of the rye fields from Dyrotz must have received dung, while others did not. Low to negative *δ*^15^N values, as seen at Dyrotz, are reported for herbs and grasses growing on forest floors and are argued to be owing to ^15^N-depleted tree litter [64]. Possibly the low *δ*^15^N rye from Dyrotz was cultivated on fields either not manured since forest clearing and therefore reflecting values of modern clearings or fields, and/or located too far from the settlement for labour-intensive cultivation.

### (d) Statistical outliers

At the archaeological sites of Oldorf, Altenwalde and Diepensee, single rye grains show high *δ*^15^N values that fall outside the range of the other grains. Such grains are also reported by Larsson *et al*. [15] for Sweden (figure 3) and appear in the unmanured recent rye from Thyrow as well. These statistical outliers may originate from plants growing in extreme ^15^N-rich places, such as close to dung heaps, or on spots in the field where domestic animals have left droppings during stubble pasturing, or where remnants of (small) animals have decayed.

### (e) On the question of crop yields

The grain yield of winter rye on sandy soils in northeastern Germany was around 1 t ha^−1 ^in the first half of the nineteenth century [65]. Körnicke [66] reports averages of 1 t ha^−1^ for all of Germany, with 0.5 t ha^−1^ for the worst and 2 t ha^−1^ for the best fields. The yields of the manured rye of Halle were around 2.7 t ha^−1^ at the turn of the twentieth century [24].

**Figure 4.**
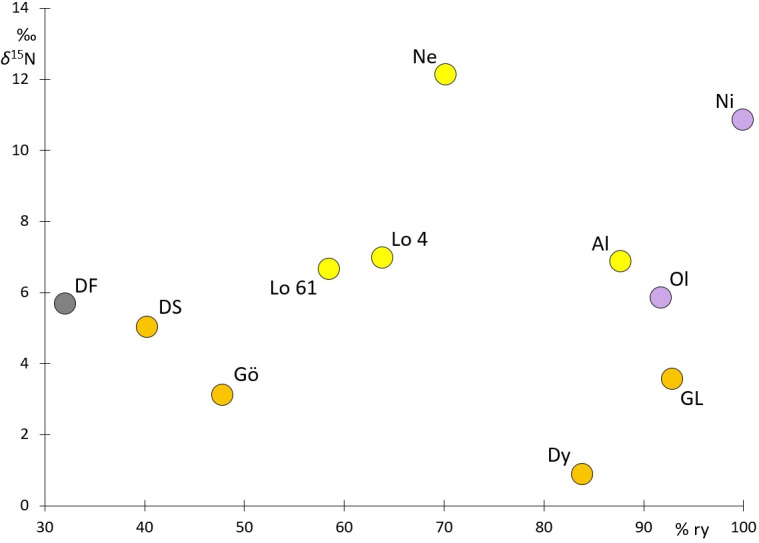
Mean relative yields (ry) and *δ*^15^N of the archaeological material (for acronyms, see table 2).

As discussed, yield amounts, ^13^C content of grains and dryness are strongly correlated. Kottmann *et al*. [58] and Krafft [67] report ry owing to water stress of up to 60−80%. Based on the mean Δ^13^C values of the archaeological samples, the ry was highest on the fields of Niens (100% ry), closely followed by Groß Lübbenau (93% ry), Oldorf (92 % ry), Altenwalde (88% ry) and Dyrotz (84 % ry) (figure 4). All other sites have lower to much lower Δ^13^C values and therefore lower ry. Δ^13^C from the Caucasus points to ry of one-third compared to Niens. The chain of effects from the intensity of manuring to increased grain *δ*^15^N and higher grain yield under different moisture regimes is not fully understood yet. It can only be speculated that, despite the ^13^C content, the more intensively manured rye from Neermoor produced a yield close to that of Altenwalde or even higher. On the other hand, owing to the higher level of manuring, the fields of Altenwalde may have been as productive as those at Groß Lübbenau. For a more detailed estimation of archaeological cereal yields in relation to water and nitrogen supply, dedicated plot experiments including ^13^C and ^15^N measurements are required. Another variable, but one that is hard to determine, is a possible difference in yields between summer and winter rye, which was approximately 25% in the nineteenth century [66] .

### (f) *δ*^34^S of ancient rye grains

**Figure 5.**
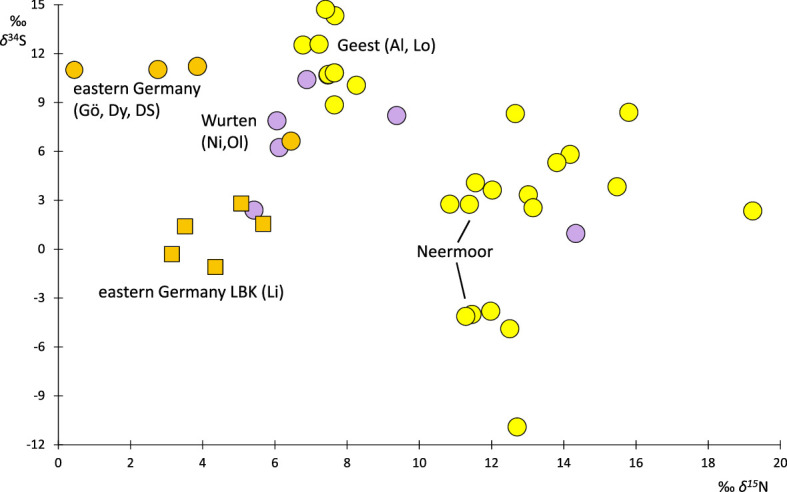
*δ*^34^S and *δ*^15^N of individual archaeobotanical grains grouped by region (eastern Germany, Geest, *Wurten*) or presented by site (Neermoor and the LBK site of Lietzow).

The soil *δ*^34^S is the result of geology and hydrology. Under wet, oxygen-deficient conditions, microbial processes strongly affect the sulfur isotopic composition [68]. Sulfur uptake happens nearly without fractionation, and plants quite closely reflect the soil *δ*^34^S. It can range from approximately −5 to 22‰ in terrestrial plants, while in ocean water, it is a constant 21‰ [54,69]. Reports on ^34^S from rye are scarce in the literature; the data published here seem to be the first on archaeobotanical material. Owing to the sample quantities needed, measurements per site are very limited and are therefore merged here for the environments *Wurten* (Oldorf, Niens), Geest (Altenwalde, Loxstedt) and eastern Germany (Göritz, Diepensee, Dyrotz). Neermoor stands out in many measures. To understand the possible palaeoecological significance of *δ*^34^S values, we also include data on Neolithic emmer (*T. dicoccum*) from Lietzow. With ranges of 8.9–14.7‰ and 6.6–11.2‰, respectively, the *δ*^34^S of rye from the Geest sites and the eastern German sites is quite similar, probably owing to them having in principle the same geological substrate as well as low water saturation of the porous soils (figure 5). The somewhat higher values for the Geest may originate from marine influence via sea spray (*δ*^34^S *ca* 21‰) and marine precipitation (*δ*^34^S *ca* 13‰) [54]. Although the *Wurten* are closer to the sea, their rye *δ*^34^S is only 1–10.4‰. These lower values may be owing to a different substrate and thus may be influenced by the manure that builds up the *Wurten* mounds.

Some of the rye from Neermoor, with a *δ*^34^S of 2.3–8.4‰, falls within the range of the *Wurten*. A *δ*^15^N of 10.9–19.3‰ indicates that the isotopes of these grains derive most probably from dung. The remaining rye from Neermoor also exhibits a relatively high, but narrower, range of *δ*^15^N (11.3–12.7‰), but a negative *δ*^34^S, of −3.8 to −11‰. The peats near Neermoor consist of fen and raised bog peats on top and became flooded by salt water with rising sea level, which enriched them with salts including sulfates. A microbial fractionation during sulfate reduction under the anaerobic conditions of the peats can lead to a *δ*^34^S of around −10‰ or even −20‰ [70]. The cutting of such peats for salt extraction became widespread from the twelfth century at the latest [71]. The use as soil improvement of fen peat layers not suitable for the extraction of salt could be a reason of the low *δ*^34^S of the rye. Nevertheless, it is possible that the dung accumulating over winter in stables or dung heaps was at least in its deeper parts saturated by animal urine and therefore anaerobic, leading to *δ*^34^S reduction, as some grains from the *Wurten* suggest.

The Neolithic emmer from Lietzow also shows a strikingly low *δ*^34^S (−1.1 to 2.8‰), especially in comparison with rye from that area. The geological substrates have the same sources, but adjacent to Lietzow, there is also a swampy area. Possibly, peat from the swamp was used as a soil improver for the fields, leading to the relatively low *δ*^34^S in the cereals. To substantiate the above assumptions and increase the value of charred grains as archaeological and palaeoecological archives, a stronger focus on *δ*^34^S is required.

## 5. Discussion

### (a) From weedy to cultivated rye

In southwestern Asia today, the many varieties of weedy rye (subsumed under *Secale cereale* ssp. *ancestrale* Zhuk. [72]) vary in their rachis traits, from fully shattering to half shattering to non-shattering. As long as mechanical weed control is weak, shattering has advantages. Grains falling to the ground can sprout and establish new plants before cereals are sown. This applies even more for winter forms, as being able to start growing directly after the harvest makes them strong competitors against cereals sown later and especially against the summer cereals of the following year. With more effective mechanical weed control involving ploughing with a mouldboard plough, this ahead strategy became a drawback and non-shattering forms of weedy rye harvested, threshed and sown together with the cereals after ploughing now had a positive selection advantage. Thus, with the dissemination of the mouldboard plough in Europe in the last centuries BCE [73], weedy rye may have turned into a weed with solid rachis as domesticated trait, depending fully on human threshing and sowing for its survival. Where drought and cold events periodically damage the cultivated cereals, farmers tolerate upcoming weedy rye, which can make up to over 40% of the grain yield in adverse years [2]. Depending on the frequency of dry years, the step to an intentional cultivation of rye may have taken quite a short time, particularly on sandy soils susceptible to dryness. On the sandy soils of eastern Germany, an experimental field cultivated with a half-and-half mixture of wheat and rye turned into a nearly pure rye field after 3 years by being sown with the resulting harvest admixture [4].

It therefore appears that, in addition to other factors [4], the invention of the mouldboard plough—together with its use on sandy soils prone to drought and, in the east, the cold, dry continental climate—may have turned weedy rye into a valuable staple crop (*Secale cereale* ssp. *cereale*) in Europe.

### (b) On the role of manuring in rye cultivation

In northwestern Germany, rye came into cultivation by the turn from the first to the second century CE at the latest [4,74,75]. The Geest around Altenwalde and Loxstedt was already a centre of cultivation in the Roman period. The accompanying weed flora indicates that in the beginning, most probably summer rye was being cultivated [74]. In that same period, rye became a co-dominating cereal in Brandenburg [36]. As indicated by the increase in typical winter weeds in archaeobotanical material and pollen records, the change to winter rye took place at the beginning of the second millennium and was accompanied by a strong expansion of area under rye cultivation [11,76,77].

Rye finds occurring occasionally on the *Wurten* along the North Sea coast in the Iron Age and becoming more frequent after the Migration period were interpreted to be an import from the Geest [28,78]. However, this interpretation is contradicted by the differences between the isotope signatures of the *Wurten* and the Geest, which indicate that the rye was more probably cultivated on the *Wurten*. Nevertheless, further analyses may provide indications also for imported cereals. The high *δ*^15^N signals from Niens and, even more so, the early manuring at Loxstedt as well as Göritz seem to contradict the idea that rye was primarily cultivated because of its low nutrient requirements. Our results from the Caucasus clearly point in the same direction. It is very likely that the rye was integrated into an existing cultivation system that already relied on manuring.

Manuring is needed to maintain high yields not only on sandy soils, where nitrogen is the most limiting nutrient for crop production [79], but also on the fertile loess soils of the Eternal Rye Experiment of Halle/Saale, where the decadal mean yield from an unmanured rye plot halved within the first four decades, from 2.3 to 1.1 t ha^−1^ [24]. Compared with crop systems with fallow years for some recovery, systems incorporating the use of manure may be much more effective, but this depends on manure availability and knowledge. In the coastal areas of the North Sea and the Baltic Sea, stables were integrated into the houses from the Late Bronze Age onwards and provided easy access to animal dung [80–82]. However, dung could also have been collected, for instance, from pastures or kraals [1,81]. In the sandy lowlands facing the North Sea, the application of dung, settlement waste and heath sods to increase the fertility of fields dates back to the first millennium BCE, with the Celtic fields [83–85]. South of the Baltic Sea, the amelioration of soil quality through human management may have started around the Bronze Age–Iron Age transition [86]. In eastern Germany, there is evidence of anthropogenic improvement of soil fertility through the application of settlement waste and possibly dung starting in the seventh century BCE [87].

Evidently, dung availability and the knowledge of its wise use were widespread, and dung manuring was practised in northern Europe long before rye cultivation began. Under certain circumstances, it was a big advantage to manure even the undemanding rye. The poor soils were one reason that the Geest was only populated by individual farmsteads in the Neolithic [88]. Increased yields through manuring probably enabled the transition to villages, with their larger populations, which took place on the Geest during Roman times [88]. At some places with wheat cultivation, including possibly at Diepensee, rye may have grown on manured fields in crop rotation with the demanding wheat. In the case of Dyrotz, rye was cultivated on poor soils that may not have been suitable for wheat. The comparatively higher rye *δ*^15^N on the Geest sands may point to farmers having had ample access to dung from the extensive cattle breeding on the rich pastures in the marshland between the site and the sea [28].

From a spatio-temporal perspective, the level of manuring of the rye shows no trend over time. Even within the oldest periods covered here, the rye from Göritz (first to fourth centuries), Georgia (fourth century), Loxstedt (fourth to sixth centuries) and Niens (seventh to eighth centuries) represent all levels, from low to intensive manuring. This applies even more for the high and late Medieval material, which exhibits the lowest (Dyrotz) as well as the highest (Neermoor) ^15^N content of all ancient material discussed here. The *δ*^15^N values of rye from outside Germany range from 5.7 to 11.4‰. Most of the sample averages and ranges are around the values measured at Thyrow for dung, of 15 t ha^−1 ^ yr^−1^. This includes the oldest material, from Georgia, as well as the youngest, from Deventer Burseplei (fifteenth century, The Netherlands) [17]. In this group, rye from a fertile loess area in Romania (RCu) [19] as well as from the poor soils of Sweden (Uppåkra house, SUh) [15] have the highest ^15^N values, and they fall completely or partially into the lower range of those from the loess soils of Halle (12 t ha^−1 ^ yr^−1^). The rye from Uppåkra house was nearly as intensively manured as the rye from Niens and Neermoor. By contrast, the rye from the nearby Uppåkra oven (SUo) exhibits a significantly lower ^15^N level [15]. Obviously, the people of the regional centre of Uppåkra consumed rye produced under very different manuring regimes. While Kanstrup *et al*. [48] found a long-term trend towards increasing manuring of barley in northern Europe over the millennia BCE, we do not observe any trend over time in manuring of rye, neither within Germany nor across Europe. The higher *δ*^15^N values from the sandy soils at the German coast compared with the inland are most probably caused by the plenty of dung from the high livestock population on the rich meadows of the German clay district.

## 6. Conclusion

Isotopic analyses (*δ*^15^N) indicate that even the early cultivation of rye in northern Europe was promoted by substantial manuring. The transformation of rye from a weed to a domesticated crop was apparently fostered by a number of technological developments [4]. In particular, the intensification of weed control through the introduction of the mouldboard plough around the transition to the Common Era may have resulted in rye traits that were, by the formation of non-shattering ears, pre-adapted to domestication. Weedy rye may have served initially as a backup crop in years of harvest failure owing to cold or drought before becoming recognized as a cereal in its own right. Rye became a dominant crop not because it was undemanding but because it was productive to integrate rye cultivation into a diverse and labour-intensive manuring system.

No discernible trend in manuring practices could be identified across the periods under consideration. It seems reasonable to posit that the use of manure was an intentional decision depending on local requirements and opportunities. The current evidence does not support the hypothesis that rye was imported from the Geest to the marshlands. It is more plausible that rye was produced locally on the elevated dwelling mounds in the marshlands. The relatively high ^15^N level of rye from the dwelling mounds and the Geest may relate to the large cattle population in the surrounding fertile marshlands and the accumulation of manure from the winter housing of these animals. Peat was possibly used as fertilizer too. The transition from the cultivation of summer rye to the permanent cultivation of winter rye during the Medieval period resulted in rye becoming the dominant cereal, until its replacement by wheat in the mid-twentieth century.

As recent experiments in rye cultivation have shown, the *δ*^15^N value in the cereal grains does not directly reflect the amount of manuring. Rather, the soil quality (sand, loess) has a major influence on the *δ*^15^N of cereal grains resulting from manuring.

The data presented here show that crop cultivation and animal husbandry were deeply intertwined and stimulated the domestication of rye. Animal dung was an important resource that, together with other organic matter, enhanced the crop yields. The resulting rise in rye production area and yields could have contributed to the advent of social change and later, towards medieval times, reinforced social hierarchy and inequality. The discovery of large quantities of rye in medieval castles, including German Neermoor and Groß Lübbenau and the Slavic Starigard, Parchim and Tornow [89–91], several churches on the Geest [92,93] and such cities as Bremen [94], indicates that the elites in northern Germany and in the Slavic area maintained their authority by stockpiling rye. The application of manure is thus one factor that contributed to the social transformation from the traditional individual farmsteads towards the emergence of village communities and towns.

Methodologically, it is evident that ³⁴S allows differentiating between sample subgroups that cannot be distinguished by ^13^C and ^15^N alone. Furthermore, *δ*³⁴S has the potential to identify processes related to hydrological processes in the widest sense, taking place in peats, dung and soils. This can point to environmental conditions and cultivation techniques. The application of triple isotope analysis to a range of crops, weeds and wild plants could enable the generation of a more comprehensive understanding of agricultural management practices at multiple scales, including sites, species, single fields, cultivation systems and the level of landscapes, as well as better identification of different sources in food webs.

Experimental cultivation may further clarify whether rye is superior to other cereals with regard to manuring. However, it should be noted that rye may have been the preferred cereal on poor soils where fertilization was not feasible owing to either a lack of manure or remoteness. Results from specific experimental plots would be beneficial for estimates of absolute crop yields in relation to water, nitrogen and soil quality.

## Data Availability

All data are included in the uploaded electronic supplementary material files [95].

## Dedication

This publication is dedicated to Prof. Dr Karl-Ernst Behre on the occasion of his 90th birthday. We would like to thank him for his fundamental work in the field of archaeobotany and far beyond.
